# The ‘cognitive behavioural model’ of chronic fatigue syndrome: Critique of a flawed model

**DOI:** 10.1177/2055102919838907

**Published:** 2019-04-23

**Authors:** Keith Geraghty, Leonard Jason, Madison Sunnquist, David Tuller, Charlotte Blease, Charles Adeniji

**Affiliations:** 1 University of Manchester, UK; 2 DePaul University, USA; 3 University of California – Berkeley, USA; 4 Harvard University, USA

**Keywords:** biopsychosocial model, chronic fatigue syndrome, cognitive behavioural model, cognitive behavioural therapy, myalgic encephalomyelitis, treatment

## Abstract

Chronic fatigue syndrome/myalgic encephalomyelitis is a debilitating illness that greatly impacts the lives of sufferers. A cognitive behavioural model attempts to explain illness onset and continuance with a hypothesis that the illness is perpetuated by patients’ irrational beliefs and avoidance behaviours. This theory underpins the promotion of cognitive behavioural therapy, a treatment that aims to change beliefs and behaviours. This article reports on a detailed review of the cognitive behavioural model. Our review finds that the model lacks high-quality evidential support, conflicts with accounts given by most patients and fails to account for accumulating biological evidence of pathological and physiological abnormalities found in patients. There is little scientific credibility in the claim that psycho-behavioural therapies are a primary treatment for this illness.

## Introduction

Myalgic encephalomyelitis (ME) is a post-infectious disease, causing lingering malaise, muscle weakness and nervous system complaints, primarily pain, cognitive dysfunction and sleep disturbance, described as far back as the 1950s (Ramsay, 1957). Chronic fatigue syndrome (CFS) is an alternative label introduced in the late 1980s to describe a syndrome of chronic unexplained fatigue (Holmes, 1988). There has been some controversy whether or not the ‘fatigue syndrome’ of CFS covers Ramsay’s ME disease. However, the two terms are often used in combination in the literature (Jason et al., 2003), thus we will use ‘ME/CFS’ in this article, or ‘CFS’ if referencing a study that only uses the term CFS. Prevalence rates vary widely across studies, but around 0.5 per cent is a commonly reported figure for adults (Nacul et al., 2011). Several diagnostic criteria have been proposed to help identify potential cases. In the United Kingdom, the National Institute for Health and Care Excellence (NICE) recommends a diagnosis after 4 months of persistent unexplained fatigue, that is not relieved by rest and results in a substantial loss of normal physical or social function (Baker and Shaw, 2007). The US Centers for Disease Control and Prevention (CDC) criteria requires a set of characteristic symptoms (Fukuda, 1994), while other criteria require the presence of post-exertional neuro-immune exhaustion (Carruthers et al., 2011).

A wide range of treatments have been tested on ME/CFS patients, ranging from drug therapies, mainly antidepressants and immunological modulators, to non-pharmacological therapies, predominantly psycho-behavioural therapies (Smith et al., 2015). Over the last two decades, cognitive behavioural therapy (CBT) and graded exercise therapy (GET) have gained prominence. Patients with ME/CFS are prescribed a modified form of CBT to challenge their illness beliefs (cognitions) and GET to increase their activity levels and diminish their alleged fear-avoidance behaviour (Sharpe, 1998; Sharpe et al., 1991). The symptoms that many patients present with, such as fatigue or pain, are posited to be ‘maintained’ by ‘dysfunctional illness beliefs’, embedded within a social and health system that rewards illness behaviours (Halligan and Aylward, 2006; Wessely, 1997). The rationale for the use of CBT and exercise therapies is linked to a so-called ‘*Cognitive Behavioural Model of CFS*’ (CBM) that is set out as a theoretical framework for illness onset and continuance (Surawy et al., 1995; Wessely et al., 1989). This CBM emerged in the early 1990s and has remained largely intact, influencing clinical guidelines for diagnosis and therapeutic approaches for ME/CFS.

Challenges to the CBM, in terms of efficacy and applicability, are scant in the literature and have not addressed the CBM in totality. Various studies have shown that patients do not possess some of the personality or behavioural characteristics deemed targets of treatment in the CBM (Song and Jason, 2005; Sunnquist and Jason, 2018). More recently, the model has come under scrutiny following publication of controversial clinical trials of CBT-GET, such as the PACE trial (Pacing, graded Activity, and Cognitive behaviour therapy: a randomised Evaluation) and the FINE trial (Fatigue Intervention by Nurses Evaluation) (Wearden et al., 2010; White et al., 2011) that sought to validate the CBM. Findings from these trials resulted in criticism that the efficacy of these treatments is poor (Geraghty and Blease, 2016; Wilshire et al., 2018). The PACE trial reported a recovery rate of 22 per cent using CBT, whereas re-analysis of data from PACE puts recovery below 10 per cent (Chalder et al., 2017; Geraghty, 2017a; Wilshire et al., 2016) – only little higher than the recovery rate observed in the de facto control group of usual medical care (Sharpe et al., 2015). Clinicians and medical philosophers argue that the CBM of ME/CFS downplays the severity of the illness, stigmatising sufferers with claims that recovery from the illness is dependent on a patient’s efforts and engagement in psychotherapy (Blease et al., 2017; Komaroff, 2015). In response, we undertake a detailed review of the ‘*cognitive behavioural model of ME/CFS*’ to assess model applicability and validity. We provide a brief description of the origins and antecedents of the CBM and its key features. We summarise how the CBM has been applied in practice, drawing on evidence from clinical trials and practice. We reveal how the model is often inconsistent, contradictory and lacking in a unifying logic. We pinpoint weaknesses in CBM theory and highlight evidence that disproves or challenges the central tenets of the CBM. Specifically, we discuss how the CBM is biased towards the psycho-social (viewing cognitions and behaviours in ME/CFS as dysfunctional rather than rational), while neglecting to account for an increasing array of evidence of biological abnormalities found in ME/CFS patients.

## The CBM of ME/CFS

CBT originated in the ground-breaking work of Beck in the 1960s and 1970s, as an experimental psychological treatment for depression (Beck, 1976). Since then, the application of CBT has widened to the treatment of anxiety, phobias, obsessive compulsive disorders and more recently, ME/CFS (Wessely et al., 1989). In the United Kingdom, in the late 1980s/early 1990s, psychiatrists proposed that Beck’s CBT could be used to treat ME/CFS. A model of CFS was proposed by psychiatry that dismissed Ramsay’s ME organic infectious disease in favour of a mostly psychogenic model of CFS. The ‘cognitive behavioural model’ of CFS is Beck’s CBT modified and applied to CFS. In this sense, the CBM of ME/CFS is distinct from Beck’s CBM for depression – and must be assessed on its own merits.

The CBM is framed around three interconnecting illness factors (‘precipitating’, ‘predisposing’ and ‘perpetuating’) – ‘3Ps’. This framework may originate from Lang et al.’s three-system model of fear maintenance and desensitisation (Lang et al., 1970). ME/CFS is viewed as an illness continued by fearful cognitions that limit patients’ activities, whereby experiences of physiological symptoms – such as pain or fatigue – reinforce unwanted cognitions and avoidance behaviours. In the CBM, the aim of treatment is to address ‘unhelpful’ cognitions and behaviours that are hypothesised to maintain the illness (Butler et al., 1991; Wessely et al., 1989). The CBM of CFS is discussed conceptually by Wessely et al. (1989) and is formulated as a theoretical model by Surawy et al. (1995). Surawy et al.’s paper draws on clinical observations from the treatment of 100 CFS patients at a hospital in Oxford, UK (Sharpe, 1993).

The CBM is also embedded within a grand biopsychosocial (BPS) model in which biological, social and psychological factors are regarded as important in understanding illness (Engel, 1977). Moss-Morris et al. (2013) state that ‘*It is unlikely that CFS can be understood through one aetiological [mechanism]. Rather it is a complex illness which is best explained in terms of a multi-factorial cognitive behavioural model*’ (p. 303). A CBM is said to capture the complexity of CFS – it connects the 3Ps within a BPS framework (as illustrated in Table 1).

**Table 1.** The biopsychosocial theoretical framework of CFS onset and continuance (ME dropped).

	Predisposing	Precipitating	Perpetuating
Biological	Age, sex, genetics	Infection, injury	Neuro-immune changes, hormonal imbalance, biochemical changes
Psychological	Childhood abuse, childhood adversity, personality traits, family history of mood disorders	Stress, traumatic events	Catastrophising, somatising, perfectionism, activity avoidance, illness beliefs
Social	Socio-economic class, social history	Life events, adversity	Social care system, illness/sickness benefits, cultural/social trends

Abbey argues that CFS patients attribute their illness to a physical cause, such as a virus; however, given clinical investigations often find no evidence of ongoing viral illness (from routing blood tests for example), such beliefs must be ‘… *illness attributions and dysfunctional automatic thoughts and cognitive distortions in patients with CFS*’ (Abbey, 1993). Physical illness attribution cognitions are a target for cognitive behaviour therapy, which aims to ‘… *optimize patients’ level of physical and psychosocial functioning*’ (Abbey, 1993). CBT seeks to reverse so-called dysfunctional beliefs. Wessely et al. (1998) argue that a belief in organic illness stops an ME/CFS patient engaging in normal activities, resulting in avoidance behaviour. Unfortunately, neither Abbey nor Wessely proffer evidence that ME/CFS patients’ beliefs are indeed dysfunctional. An assumption is simply made that if no standard underlying disease pathology can be found to account for ME/CFS patients’ symptoms, a belief in disease or infection on the part of the ME/CFS sufferer is irrational. We shall show later in this article that considerable evidence of abnormal biology exists in ME/CFS – rendering the dysfunctional beliefs theory misguided and inaccurate.

Abbey (1993) goes on to write, ‘*The CBT programme is predicated on an* “*alternative view*” *of CFS which posits that rest and avoidance, which may have been adaptive during the acute phase of the illness, are deleterious and perpetuate disability later in its course*’. Abbey also postulates that CBT helps with ‘learned helplessness’, a concept discussed by Seligman (1975) to describe a feeling of losing control of thought and one’s life and falling into a pit of depression (Maier and Seligman, 1976). CFS is viewed as analogous to depression and is said to be linked to somatic and psychological distress (Wessely et al., 1996). Proponents of the CBM write,

cognitive behavioural models propose that beliefs about the unacceptability of experiencing or expressing negative thoughts and emotions can play a central role in the development and maintenance of clinical problems and can be associated with a poorer prognosis or treatment outcome. (Rimes and Chalder, 2010)

Here, a new layer of theory is introduced, that ME/CFS is somehow related to a problem with expressing emotion. CBT aims to help patients consider other, less threatening explanations for their symptoms and pulls the victim out of a downward spiral of depressive thinking, somatising and catastrophising (Sharpe, 2007; Wessely et al., 1998). The aim of therapy is reattribution of beliefs and reversal of depressive-anxious thoughts and avoidance behaviours.

We note at this point, that it is clinicians, mostly psychiatrists, acting as the arbitrator of what is a ‘useful belief’ or a ‘dysfunctional belief’ and what ‘alternative explanations’ should be given to ME/CFS patients. Such strategies may have merit in illnesses such as obsessive compulsive disorder, where a patient might think that eating in a cafe is going to cause great harm due to exposure to germs – the patient’s fears are challenged with the rational argument that eating in a cafe is unlikely to cause any harm. However, in ME/CFS, the application of this approach lacks a rational alternative explanation. Most patients report that their illness started after an infection and an increasing body of literature links ME/CFS to infections and immune alterations post infection (we discuss later in article). The CBM aims to change patients’ unhelpful thoughts and experiences of symptoms (pain, fatigue and malaise) with supplanted alternatives. ‘I am sick, I think its related to an infection, I am tired’ (patient) to ‘you do not have an organic disease, resting is harmful, you can do activities’ (CBT therapist).

**Figure 1.**
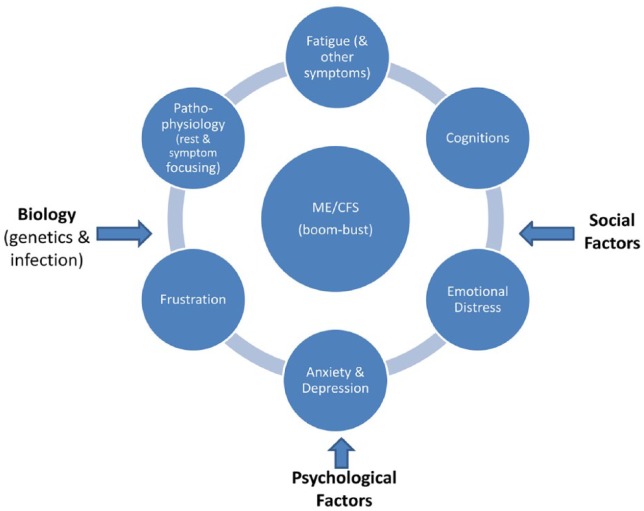
The CBM of ME/CFS.

Sharpe (2007, citing Wessely et al., 1989) concedes that the theory behind the use of CBT in CFS is weak: ‘… *treatment is plausible, but lacks a theoretical rationale. It is possible to construct a hypothetical model by assuming that the aforementioned factors interact in self-perpetuating vicious circles*’. The notion of ‘a cycle’ is repeated throughout the CBM literature. Butler et al. (1991) state that ‘*The result is a vicious circle of symptoms, avoidance, fatigue, demoralisation and depression-the clinical picture of CFS*’. Deary et al. (2007) state that ‘*The sine qua non of any CBM is a vicious circle, the hypothesis that a self-perpetuating interaction between different domains maintains symptoms, distress and disability*’ (p. 782). This rudimentary hypothesised cycle is outlined by Surawy et al. (1995) and again by Sharpe (2007) and others (Harvey and Wessely, 2009) – that CFS may begin with a viral infection, which leads to symptoms such as fatigue or pain, which cause a sufferer to rest and become fearful of activity (perpetuated by beliefs about organic illness), whereby avoidance behaviours lead to anxiety and depression, which exacerbate physiological and mental deconditioning (model derived by Sharpe, 1993, 2007). This cyclical model is similar to the fear-avoidance model of chronic pain (Crombez et al., 2012). Figure 1 represents an illustration of the CBM and the factors and components of the model. An assertion is made that by addressing one or more factors, such as unhelpful cognitions/behaviours, catastrophising, or anxiety and fear avoidance, CBT is able to halt the perpetuation of the illness (Petrie et al., 1995).

Social influences (Figure 1) are another layer in the model narrative said to sustain CFS: the rewards of the sick-role, social care benefits and peer support in sickness (Halligan and Aylward, 2006). The assertion is made that a return to normal activity would result in the loss of such social benefits and that ME/CFS sufferers remain sick to avail sick-role benefits (Becker, 1952). Again, a speculative narrative is proposed with little evidential support. Overall, the CBM impacts ME/CFS sufferers’ access to disability/social and medical support (Sharpe and Wessely, 1998; Wessely, 1997; Wessely et al., 1998). The CBM is utilised as a theoretical framework for both the testing of CBT-GET in clinical trials and as a rationale for treatment of ME/CFS in clinical practice.

## Limited model validation

Vercoulen et al. (1998) used structural equation modelling to generate a CBM of CFS with factors such as beliefs about the disease being somatic linked with fatigue outcomes. Vercoulen et al.’s (1998) model characterises patients with CFS as having insufficient motivation for physical activity or recovery, lacking an internal locus of control, and maintaining a self-defeating preoccupation with symptoms (Vercoulen et al., 1998). This model is almost identical to that of Surawy et al. (1995) and has largely remained intact up to the present day, as the basic rationale behind the use of CBT in the treatment of ME/CFS (White et al., 2007). Success in bringing about improvement in symptoms, or full recovery, is said to demonstrate the explanatory power of the CBM (Sharpe, 1998). However, as we detail below, the evidence-base for CBT and GET in ME/CFS is highly contested and problematic. Researchers have also sought to validate the CBM using mediation analysis, a statistical method to test how a mediating variable transmits the effect of an independent variable onto a dependent variable. Stahl et al. (2014) sought to validate CBT efficacy in CFS by testing the role of ‘fearful cognitions’ as a mediator between avoidance behaviour and illness outcomes (e.g. fatigue). While such studies appear to support the hypothesis that ‘illness beliefs’ mediate fatigue in CFS, it is important to remember that mediation analysis is a correlation test that does not prove causality. Stahl et al. (2014) concede that the positive effects that they observed using CBT could have been brought about by other factors or treatments. Essentially, any type of one-to-one talk therapy might reduce symptomology in ME/CFS, given that sufferers experience distress, anxiety, depression and social isolation. Here, any success using CBT in ME/CFS may result from general treatment effect (Geraghty and Blease, 2018), rather than cognitive or behavioural restructuring.

Song and Jason (2005) attempted to validate Vercoulen et al.’s (1998) CBM in a US community sample and found that the model fit better for individuals with fatigue from psychiatric disorders but not for patients with CFS. Song and Jason found little support for the idea that ME/CFS is an illness maintained by sufferers’ beliefs. Jason et al. (2007) observed improvements in quality-of-life scores following CBT in CFS, but noted in a later study (Jason et al., 2007, 2008) that ‘improvers’ may be quite different in profile from ‘non-improvers’, particularly in immune profiles. ME/CFS is difficult to diagnose, and clinical trials of CBT for ME/CFS often include patients with psychiatric conditions who may respond positively to psychotherapy, whereas sufferers with post-infectious profiles often fail to respond. In the study of Deale and Wessely (2001), 82 per cent of a group of CFS patients referred to a UK hospital-based specialist CFS clinic reported being prescribed antidepressants. Jason et al. (2009) found that 38 per cent of patients with major depressive disorder were misclassified as having CFS. Essentially, many ME/CFS studies are vulnerable to illness misclassification, rendering evidence from these studies unreliable (Nacul et al., 2017).

## Problematic evidence from clinical trials and practice

Proponents of the CBM consistently argue that the model is validated via the success of CBT and GET in randomised controlled trials and clinical practice. It would be impossible to critique all CBT trial evidence within the confines of this article, thus we point readers to reviews of the field (Rimbaut et al., 2016) and systematic reviews (Larun et al., 2016; Price et al., 2008) that appear to show, prima facie, that CBT and GET outperform other treatments for ME/CFS (mostly usual care) with small-to-modest effect sizes. However, this evidence is increasingly contested by re-analysis of data from clinical trials (Geraghty et al., 2017; Wilshire et al., 2018) and meta-reviews (Vink and Vink-Niese, 2018).

Many patients seen in CFS treatment studies appear to be drawn from psychiatric centres with high rates of psychiatric morbidity. In the study of Wessely and Powell (1989), 22 (47%) patients met the criteria for major depressive disorder. The inclusion of patients with mental health complaints that might explain their presenting fatigue is a contamination issue that persists to this day in clinical trials of CBT treatment for ME/CFS (Taylor et al., 2003). In Wessely and Powell’s work, approximately half of patients with CFS were indistinguishable from the control patients with psychiatric disorders, except for illness attribution and CFS diagnosis. Butler et al. (1991) conducted a study using CBT on CFS outpatients at the National Hospital for Nervous Diseases in London (50 patients received an average of 7.5 hours CBT). Butler et al. reported that CBT led to substantial reductions in fatigue, mood problems and psychiatric complaints (60% of patients improved). However, 18 patients (36%) rejected treatment at recruitment and 5 dropped out mid-way, leaving a strong bias towards improvers.

The first, large randomised trial of immune therapy and CBT for patients with CFS in Australia found no benefit for CBT (alone or in combination) over nonspecific treatment regimens (Lloyd et al., 1993). Sharpe et al. (1996) recruited 60 CFS patients to a randomised controlled trial (RCT) in Oxford and reported that almost three quarters of patients given CBT improved versus one quarter in standard medical care. Around the same time, Wessely conducted an RCT with CFS patients, 30 given CBT and 30 given relaxation therapy (Deale et al., 1997). Astonishingly, 70 per cent in the CBT arm (19 of 27) went from bed-ridden levels of reported physical health (mean score of 25 on 36-Item Short Form Health Survey (SF-36) questionnaire) to near-perfect physical health (mean score: 70+) post treatment. Deale et al. (1997) reported that CBT changed physical function from an average baseline SF-36 score of 24.7 to an average score as high as 85.1 for improvers. However, 20 out of 30 participants in the CBT arm reported present or past psychiatric illness, including major depressive disorder (Deale et al., 1997). Recall, Beck designed CBT as a treatment for depression. Review of this study suggests that many patients were likely depressed patients given a CFS diagnosis using the Oxford criteria (Sharpe et al., 1991).

After the year 2000, the number of clinical trials of psycho-behavioural treatments for ME/CFS accelerated. Moss-Morris et al. (2005) observed a greater decrease in self-rated fatigue using GET in a trial of GET on 25 CFS patients and 24 CFS controls given standard care. However, SF-36 physical health scores differed little between groups at follow-up and many CFS patients refused to undertake physiological exercise tests (10 patients reported that testing harmed them and 5 could not reach maximal effort in testing; Moss-Morris et al., 2005). Wiborg et al. (2010) observed that reduced fatigue in three randomised trials of CBT was not mirrored by an increase in physical activity as measured objectively with actometers. Essentially, in CBT-GET RCTs that report evidence of improvement in subjective self-reported fatigue, there is often little or no evidence of objective improvement in physical fitness or function.

The largest clinical trial of CBT-GET in ME/CFS, the PACE trial, reported a 60-per cent-plus improvement rate and a 22-per cent recovery rate using CBT plus standard medical care, compared with just 7 per cent recovery for standard medical care only (Sharpe et al., 2015; White et al., 2011). This trial has attracted criticism after it was discovered that the authors deviated from their published trial protocol and lowered key measurement criteria mid-trial (Geraghty, 2017b). Re-analysis using the published trial protocol reveals a recovery rate for CBT closer to 7 per cent (not the 22% reported; Geraghty, 2017a; Wilshire et al., 2016). The PACE trial also demonstrated that little improvement in objective tests of physical function and the size of between-group differences (CBT-GET vs pacing therapy or standard medical care) was not sustained at 2-year follow-up (Chalder et al., 2015; Geraghty, 2017a; Sharpe et al., 2015). Interestingly, Wessely et al.’s 1990s’ RCT reported a 50-per cent recovery rate at trial end, yet only a 26-per cent recovery rate at 5-year follow-up and most of the patients classed as ‘recovered’ continued to report considerable problems with fatigue (Deale et al., 2001). A sister trial of the PACE trial, the FINE trial, found no substantive benefits over standard care using home-based nurse-provided CBT-GET in CFS patients at 70 weeks (Wearden et al., 2010). Across RCTs, recovery rates are inconsistent, do not mirror objective scoring of improvement and fall away over the long term.

Outside the confines of carefully managed clinical trials, data from UK specialist CBT National Health Service (NHS) clinics do little to support the use of CBT-GET in ME/CFS. In one recent study, 5.7 per cent of CFS patients reported no longer having the illness after NHS treatment ends (Collin and Crawley, 2017). This equates to 94.3 per cent of patients sent to CBT centres reporting ME/CFS status post treatment. Why are most ME/CFS patients not recovering using CBT or GET? A systematic review of prognosis in CFS finds a full recovery rate of just 5 per cent (Cairns and Hotopf, 2005). It is important to note that in clinical trials and practice, studies involve ambulatory ME/CFS patients well enough to attend full courses of treatment (milder cases) – while those bedbound or housebound with moderate-to-severe ME/CFS are absent. Any modest reported benefits using CBT or GET are not generalisable to the whole ME/CFS population. The FINE trial sought to remedy this limitation by testing CBT-GET provision in the homes of CFS patients, but failed to show significant benefits over usual care at follow-up (Wearden et al., 2010). There are also a range of biases in CBT-GET trials that influence results, such as design biases, therapy effects or expectancy effects – discussed elsewhere (Geraghty and Blease, 2018). Clearly, this problematic evidence-base does little to validate the CBM of ME/CFS.

## Failure to rationalise evidence of biological dysfunction (pathology)

The CBM of ME/CFS inextricably fails to explain the origins and impact of symptoms specific to ME/CFS; the commonest symptoms reported in the illness are pain, fatigue, sleep disturbance and cognitive dysfunction. In the CBM, pain is theorised to be the result of patients somatising or overly focusing on bodily sensations (Lane et al., 1991), whereas studies of pain in ME/CFS reveal that patients experience widespread muscular, joint pain and headaches (Marshall et al., 2010). Meeus and Nijs (2007) offer an explanation that pain in ME/CFS might originate from increased central neuronal responsiveness. The authors speculate that this mechanism is triggered by infection, leading to sensitisation of the spinal cord (including wind-up or temporal summation, dysregulated descending inhibitory pathways and upregulated facilitatory modulation) in response to repetitive noxious stimuli that cause an increase in electrical discharges in the dorsal horn. In addition, inhibitory modulation may be impaired (Meeus and Nijs, 2007). Here, pain is the result of insult in the central nervous system and post-infection pathophysiological responses (Underhill, 2015).

Fatigue after exertion, a cardinal symptom of ME/CFS, is said to be associated with ‘too much rest after the initial infection stage’ by CBM promoters (Moss-Morris et al., 2013; Sharpe, 1998). The patient is said to bring about ‘physiological deconditioning’ by avoiding activity (Browne and Chalder, 2006; Surawy et al., 1995). However, this view is not evidence-based but merely hypothesised via a narrative. Moss-Morris (2005) writes,

The common response to a physical illness is rest. However, reduced activity conflicts with achievement orientation and may result in bursts of activity in an attempt to meet expectations. These periodic bursts of activity inevitably exacerbate symptoms and result in failure, which further reinforces the belief that they have a serious illness. As time goes by, efforts to meet previous standards of achievement are abandoned and patients become increasingly preoccupied with their symptoms and illness. This results in chronic disability and the belief that one has an ongoing incurable illness which may be eventually diagnosed as CFS. (p. 224)

Proponents of the CBM also assert the notion of a ‘boom-bust cycle’ or ‘all or nothing behaviour’ to account for patients’ reports of symptom flare following activity (Deary et al., 2007). Many ME/CFS sufferers experience post-exertional malaise (PEM), a worsening of symptoms following activity caused by physiological and cellular changes (Chu et al., 2018), including activation of immune cytokines and increases in cellular lactate levels (Twisk, 2015). Researchers studying PEM state that this response cannot be explained by physiological deconditioning alone (Cook et al., 2017). A series of studies of exercise capacity, including cardiopulmonary exercise test (CPET), reveal the pathophysiology underlying the PEM in ME/CFS patients is a characteristic of the disease, not seen in healthy controls or sedentary controls (Stevens et al., 2018). Such evidence is often ignored by CBM authors. Patients with ME/CFS are 10.4 times more likely to experience PEM compared with healthy controls (Brown and Jason, 2018). In contrast, the CBM interprets patients’ reduction in activity as ‘fear avoidance behaviour’ (Browne and Chalder, 2006; Chalder et al., 2017) and does not consider patients’ avoidance of activity as a rational response to PEM (Sharpe, 1998; Wessely et al., 1989; White et al., 2007). Yet, evidence of biological dysfunction (loss of homeostasis) and neuro-immune events, in many ME/CFS sufferers (Komaroff et al., 2018; Monro and Puri, 2018), aligns with patients’ accounts of the illness.

The US Institute of Medicine (IOM) in 2015 conducted an extensive review of the literature, including expert testimonies from the most prominent US ME/CFS experts and concluded that ME/CFS is a ‘serious biological illness’. Similarly, the US National Institutes of Health (NIH) concluded that ME/CFS is ‘not a primary psychological disease’ (Green et al., 2015). Both the IOM and NIH point to a range of biological/physiological abnormalities observed in patients, including immune dysfunction, cardiac dysfunction and neuro-cognitive deficits. Notable studies include findings of biological markers indicative of energy metabolism dysfunction (Armstrong et al., 2012; Fluge et al., 2016; Germain et al., 2017; Naviaux et al., 2016) that partly explains the origins of fatigue experienced by sufferers; or findings of neurological inflammatory markers and gross inflammation and anatomical changes (Finkelmeyer et al., 2018; Lange et al., 1999; Nakatomi et al., 2014; Shan et al., 2016); and evidence of systemic immune activation (Brenu et al., 2011; Hornig et al., 2016; Landi et al., 2016; Montoya et al., 2017; Smylie et al., 2013). This small fraction of building evidence points to a ‘neuro-immune-cellular dysfunction model’ of ME/CFS that explains the symptoms reported by patients: pain, fatigue, cognitive dysfunction and malaise after effort.

A major limitation of the CBM is that it does not fully consider the role of pathogens in ME/CFS. For instance, research has shown that ME/CFS is linked with exposure to Epstein–Barr virus (EBV), Coxsackie B, human herpes virus (HHV) 6 and 7, and Coxiella burnetii (Katafuchi et al., 2005; Underhill, 2015; Wuest et al., 2014). Chia and Chia (2008) proposed a link between ME/CFS and enterovirus infection in the stomach after 135 of 165 CFS patient (82%) biopsies stained positive for VP1 within parietal cells versus just 7 of 34 (20%) healthy controls. Clearly, infectious agents are a risk factor in ME/CFS onset. The CBM accepts that viruses might trigger ME/CFS initiation, but the CBM fails to consider that infections and immune/cellular disruption post infection may account for the symptoms reported in ME/CFS. ‘*Viruses may not be either necessary nor sufficient for the development of CFS*’ (Wessely and Powell, 1989). In fact, CBM promoters suggest that ME/CFS patients do not have organic illness or biological dysfunction caused by infection, but rather irrational cognitions or illness attributions. However, outside of ME/CFS, it is known that common HHVs cause a range of acute and chronic illnesses, including encephalitis/meningitis (herpes simplex virus type 1 (HSV-1) and HSV-2, and HHV-6), shingles, chicken pox (varicella zoster), hearing loss, mononucleosis and post-viral fatigue syndrome (EBV), Kaposi’s sarcoma (HHV-8), and atherosclerosis, hearing loss, mental retardation, retinitis (human cytomegalovirus (HMCV)) and multiple sclerosis (HHV-6 and EBV) (De Bolle et al., 2005; Wuest et al., 2014). Montoya et al. (2017) have implicated herpes viruses as a likely trigger for the inflammatory immune profiles observed in ME/CFS patients. Despite growing evidence of biological irregularities in ME/CFS patients and almost exclusive accounts from patients that the illness starts after an infection, CBM proponents continue to ignore evidence that does not support their preferred model.

## Inconsistency in CBM and contradictory evidence

Wessely (2001), an architect of the CBM of ME/CFS and the CBT-GET treatment paradigm, writes in a 2001 *JAMA* editorial that,

‘… even though these interventions appear effective, the evidence is based on a small number of studies and neither approach is remotely curative’… and that ‘these interventions are not the answer to CFS but, based on currently available evidence, seem to be among the best available options’.

Wessely affirms that CBT and GET are not curative treatments but are the only viable options in the absence of other treatments. This statement is a stark contradiction to much of the discourse we observed in the literature, where it is often suggested that CBT-GET treatments are curative (such as in the PACE trial), where a patient was deemed ‘recovered’ if they no longer met specified criteria (White et al., 2007). However, the low level of recovery observed in clinical trials and clinical practice counters claims that the illness can be cured using psycho-behavioural therapies.

In a 2010 paper titled ‘The central role of cognitive processes in the perpetuation of chronic fatigue syndrome’, Knoop et al. (2010) write, ‘*The problem with all models is that they are not based on longitudinal data … So, although the cognitive behavioral models of CFS helped in understanding CFS, they lack in specificity and empirical foundation*’ (p. 490). An inspection of the major psycho-social factors said to perpetuate ME/CFS in the CBM reveals significant inconsistent and contradictory evidence. For example, in a prospective cohort study in New Zealand following patients with glandular fever (EBV) to see which ones developed CFS (Moss-Morris et al., 2011), a list of 13–14 cognitive behavioural risk factors was investigated. At 6 months (CFS status), 17 EBV sufferers were given a CFS diagnosis from a cohort of 217. The study showed that many cognitive behavioural factors were not associated with CFS onset. Major factors, such as ‘patients limiting activity’, ‘perfectionism’ or ‘holding beliefs about infection’ showed no risk association (Moss-Morris, 2005) and for those variables with positive correlations, such as ‘all-or-nothing behaviour’, odds ratios remained low. However, the paper abstract states that ‘*The findings from this study provide support for the cognitive behavioural model and a good basis for developing prevention and early intervention strategies for CFS* [CBT]’ (Moss-Morris et al., 2011). The study also has major shortcomings. In total, 17 EBV patients were classed as CFS with an average age of 19 years. Many of the constructs studied were devised by the authors. This study, based on a small non-clinically diagnosed sample of adolescents with linked EBV, does not offer ‘good evidence’ of the predictive value of psycho-social factors.

We observe that CBM proponents iterate a role for psychological factors, even where there is contradictory evidence. For example, Moss-Morris et al. found no association with perfectionism in CFS onset and Wessely found that perfectionism did not play a role in CFS (Wood and Wessely, 1999), yet ‘perfectionism’ is claimed to be a factor in CFS maintenance (Browne and Chalder, 2006). A study of personality disorder rates among CFS patients in Finland found no difference between rates among CFS sufferers (*n* = 50) and healthy controls (*n* = 50) (Courjaret et al., 2009). A case-control study tested whether patients with CFS have exercise phobia (fear of activity), by measuring anxiety-related physiological and psychological reactions to ordinary activity and exercise (Gallagher et al., 2005). A total of 42 CFS patients were compared with 42 controls with no illness but sedentary behaviour. CFS sufferers had significantly more fatigue, sleep disturbance, anxiety and depression, compared with controls at baseline. Results revealed that CFS patients did not have exercise phobia – there was no increase in symptomatic anxiety. The authors write, ‘… *there was no more anticipatory avoidant behaviour in CFS patients compared with healthy controls*’ (Gallagher et al., 2005: 371–372). The authors go on to state that,

As both groups were equally sedentary and inactive, this suggests that fatigue was not caused by current levels of inactivity. The results from the psychological measures lend support to some of the perpetuating factors suggested by the cognitive–behavioural model for CFS … (p. 371)

The authors do not specify what ‘perpetuating factors’ remain viable in their own study. Here, we evidence the authors subjectively interpret data to support the CBM in the face of contradictory evidence. What is remarkable is that White et al. proposed avoidance behaviour due to fear of exercise as the theoretical basis of an early RCT of GET in the 1990s (Fulcher and White, 1997, 1998) and as a theoretical basis for the PACE trial (White et al., 2007). We find numerous examples of confirmation bias and selective interpretation of evidence across the CBM literature. There is a tendency to search out evidence to support the theory underpinning the CBM of ME/CFS while contradictory or conflicting evidence is often ignored.

The imputation of a behaviourist rationale in the CBM is beautifully exemplified in a study by Sharpe et al. of 166 patients followed-up after being seen at an Oxford hospital infectious disease clinic. Sharpe et al. (1992) identified a number of variables that correlated with functional impairment: ‘belief in a viral illness’, ‘membership of a patient ME group’, ‘emotional disorder’ and ‘alcohol avoidance’. Sharpe et al. speculated that these factors are important in explaining ‘perpetuation of CFS’, but also conceded that the direction of causality might be in reverse. Essentially, being more ill for a longer period might influence patients to join a support group, feel depressed, avoid alcohol or hold a belief of an underlying illness. This encapsulates the selective and narrative nature of the CBM, where inferences are made from associations between variables that often have alternative explanations. In the CBM, speculative reasoning (that holding beliefs of a physical illness increases the risk of ME/CFS onset) is postulated over the more obvious reasoning (that ME/CFS patients who suffer chronic ill health after an infection believe they have a physical illness). Wessely (1994) references Sharpe et al.’s study as good evidence that psycho-behavioural factors perpetuate CFS. Here, minor correlations are used to support a dogmatic CBM. One study with weak evidence is used to support other studies with weak evidence. We identified numerous examples of this practice across the CBM literature.

## Discussion

The ‘cognitive behavioural model’ of ME/CFS is premised on a theory that patients hold irrational and dysfunctional beliefs/cognitions that influence behaviours and illness continuance. The CBM is a representational narrative model of multiple constituent discrete parts that are said to have some explanatory, predictive and therapeutic values. Essentially, the CBM asserts that certain predisposing factors increase the risk of developing ME/CFS, then a precipitating factor or event, such as an infection, initiates the illness and later a host of psycho-social factors are involved in maintenance of ME/CFS. We find that this model narrative is contested by most patients (Spandler and Allen, 2017) – a clear warning light. In addition, research evidence continues to refute the model. We outlined a range of inconsistencies and contradictions that expose the CBM as weak and incoherent. For instance, Surawy et al.’s (1995) or Harvey et al.’s (2008) diagrammatic representations of the model offer no explanation of what specific pathogens trigger ME/CFS or how such infections cause ME/CFS symptoms, other than to suggest the sufferer wrongly attributes their symptoms to physical illness and alters behaviours in response to this belief. The CBM fails to explain why many ME/CFS sufferers experience muscle pain, orthostatic intolerance and cognitive deficits. Overly resting and somatising is an explanation offered by the CBM, with little evidential support. It is noteworthy that the fear-avoidance model of chronic pain has been criticised for lack of empirical support (Wideman et al., 2013). Interestingly, in the field of neuro-psychiatry, depression is now linked to neuro-inflammation (Miller et al., 2009) and neurological diseases, such as multiple sclerosis, are also linked with viral infections such as EBV (Pender et al., 2018).

There is a malign discourse within the CBM that implies that patients no longer correctly interpret their bodily feelings. The CBM treatment approach aims to coerce sufferers into accepting an ‘alternative explanation’ for their symptoms (Abbey, 1993) that nothing is physically wrong and that symptoms have no link to biological disease. This view is discredited by international research that finds an array of biological abnormalities in ME/CFS. Such research strongly points to immune system changes and neurological inflammation as being particularly salient (Komaroff et al., 2018; Montoya et al., 2017; Tomas et al., 2017). ME/CFS might now be considered a post-infectious neuro-immune disease, with parallels to lupus or multiple sclerosis. Patients’ beliefs about their symptoms are well-founded and logical.

Patients’ behavioural adaptations, such as resting as a result of fatigue, weakness and pain, are exactly the type of responses we might expect in chronic illness. The CBM theory that ME/CFS is perpetuated by excessive rest is not evidence-based and is contradicted by a linked CBM theory that sufferers over-do things (in a boom-bust cycle). A more rational explanation is that sufferers will continually test their limits of ability and will adjust accordingly, while attempting to revert to normal levels of activity. The stress, anxiety and depression detected in ME/CFS patients is likely related to a combination of brain and central nervous system inflammation and physical distress (symptoms of pain) and frustration at not being able to return to work or social activities (social isolation). We also suggest that lack of recognition of patients’ complaints by medical professionals adds to their distress (Blease et al., 2017).

Biological studies of ME/CFS may lead to new routes to treatment that target biological abnormalities (Younger et al., 2014), while the CBM continues to only support CBT and GET as a treatment approach. We have shown above that the success of these treatments is low and may be contaminated by the inclusion of patients with non-ME/CFS in clinical trials. Energy Envelope Theory suggests that ME/CFS sufferers who expend more energy than they have available (go outside of their limited energy envelope) experience greater fatigue and impairment (Jason et al., 2013). Jason et al. suggest careful pacing as an appropriate treatment approach in ME/CFS. Some researchers have had modest success using pacing approaches (Pesek et al., 2000), showing that alternative approaches are able to produce modest benefits without the need for belief modification.

One might assume that if the CBM accurately captures the realities of ME/CFS with logic, reason and precision, patient advocacy groups would support the model and associated treatments; however, this is not the case. Patient organisations are strongly opposed to CBT and GET as treatments for ME/CFS. Wessely (1994) and others address patients’ opposition to CBT-GET by suggesting that this is an example of anti-psychiatry sentiment and the stigma of mental health illness. However, Wood and Wessely (1999) found no evidence to support this view in one study. In contrast, a review of patient survey evidence spanning 15 years by Geraghty et al. found between 8 and 35 per cent of patients report some benefit using CBT, while 65–92 per cent report no benefit, and between 54 and 74 per cent report graded exercise to be detrimental (Geraghty et al., 2017). In a study by Deale and Wessely (2001), surveying the views of 68 ME/CFS patients referred to their CFS clinic, almost all patients expressed a view their illness had a physical cause and over half also stated they had been given a psychiatric explanation of their illness that was ‘unacceptable to them’. Clearly, patients are rejecting the CBM and report little benefit using either CBT or GET.

There is growing concern that CBT and GET are not benign medical interventions but are therapies that generate a range of iatrogenic/harmful outcomes for ME/CFS sufferers. The PACE trial found no significant evidence of serious adverse effects using CBT-GET to treat CFS (Dougall et al., 2014). However, clinical trials such as PACE define serious adverse events to include death, hospitalisations or significant deterioration, while new symptoms are considered non-serious adverse events (White et al., 2007). In contrast, a detailed review of harms by Kindlon (2011) suggests that 20 per cent of patients with CFS report adverse reactions to CBT. Geraghty and Blease (2016) highlight how harms may be more nuanced in psychotherapy. For example, if an ME/CFS patient fails to improve following CBT, they may erroneously blame themselves for this failure, particularly if a CBT therapist postulates that success is dependent on commitment to therapy. It is well documented that clinical trials of psychotherapies often fail to investigate adverse outcomes.

The CBM put forward by Wessely, Surawy, Sharpe, Chalder, Moss-Morris and others represents their speculative theories about ME/CFS pathogenesis and continuance. The CBM might be considered an ‘idealized-narrative model’ that attempts to simplify complex observations. However, it is rather obvious that all biological factors, sociological factors and psychological factors are not considered in this model – all genetic or epigenetic factors for example. The CBM appears to rest on an assumption (akin to the general systems theory of von Bertalanffy) that everything interacts. We see this eclectic grand theory in the BPS model of ME/CFS using the 3Ps framework. However, Ghamei (2010) articulates how such eclectic models suffer from being unable to define ‘saliency’ – what is most important to understand a disease. In the CBM, we find a narrow set of mainly psychological factors studied and this small number of factors is said to validate the model. As we have shown in this article, many of the factors proposed in the CBM are either not evidence-based or have weak evidential support. In addition, the role of biological dysfunction is all but ignored within the CBM, relegated to a ‘trigger event’ – rather than a major perpetuating factor. This is a fatal flaw of the CBM; it sets itself up as a model to capture complexity (the bio-psycho-social), but then rests on a subjective narrative of a small number of factors (psycho), with limited explanation for their interactions, while the existence of considerable contradictory evidence is not addressed or ignored.

In contemporary logic, a model is a structure that makes all sentences of a theory true (Hodges, 1997). The CBM is one explanation of ME/CFS – it is a stylised description of a relevant target system (Achinstein, 1968) along the lines of Cartwright’s so-called ‘simulacrum account of explanation’, which is a model to frame a theory of an explanation sought (Frigg and Hartmann, 2006). The CBM is based mostly on speculation. Far too much contradictory evidence exists for the model to be held as credible. McLauren’s (1998) paper on the flaws of the BPS model tackles the issue of how flawed models misinform practice. We believe the CBM proffers an inaccurate account of ME/CFS that misinforms clinicians, therapists and patients. A model should represent as closely as possible the subject illness under investigation. Where it does not, or is easily debunked, alternative models need to be explored. The CBM of ME/CFS is fundamentally flawed and should be abandoned as an explanatory-treatment model.

## Conclusion

In this article, we reviewed the CBM of ME/CFS. This model is often cited in the literature as a model to guide clinical practice and treatment of this illness. We find this model to be primarily an idealised narrative model. It exists as a dogmatic model favoured by model promoters. Our review exposes stark weaknesses, inconsistencies and contradictions, both in its theoretical underpinnings and the research said to prove model validity. Our findings suggest the CBM is not fit for purpose, as it poorly reflects the accounts given by patients and it ignores the wealth of evidence showing biological, immune and neurological dysfunction in ME/CFS. Given that the CBM is cited as the basis for CBT and GET interventions, there is an urgent need for clinicians, therapists and health providers to review this treatment paradigm. Our findings help explain why so many patients reject psychotherapy. An alternative model should be formulated to better explain the biological factors that predispose, precipitate and perpetuate the illness. An explanatory model needs to closely resemble illness pathogenesis and provide logic-driven linkages between factors, including patients’ symptoms and illness behaviours.
